# Ants manage polyol production to fight fungal antagonists

**DOI:** 10.1128/spectrum.02189-24

**Published:** 2025-05-14

**Authors:** Diego Santana Assis, Sérgio Kakazu, Mateus Oliveira da Cruz, Raphael Affonso Pereira da Silva, Vitor Rodrigues Marin, Milene Ferro, Daiane Cristina Sass, Andre Rodrigues, Maurício Bacci

**Affiliations:** 1 Department of General and Applied Biology, Institute of Biosciences, São Paulo State University (UNESP), Rio Claro, São Paulo, Brazil; Connecticut Agricultural Experiment Station, New Haven, Connecticut, USA

**Keywords:** *Acromyrmex*, *Atta*, *Attina*, insect infection, insect nutrition, *Leucoagaricus*, *Leucocoprinus*, mannitol, *Mycocepurus*, SDG 15 life on land

## Abstract

The attinas, ants of the *Attina* subtribe (Formicidae: Myrmicinae), colonize vast areas in the American continent. This impressive fitness depends on culturing mutualistic fungi in their “fungus gardens” to digest biomass. *Atta bisphaerica*, a derived leafcutter attina, degrades plant biomass into polyols in their fungus gardens. The proposed strategic use of polyols to accumulate energy and preserve digestive fungal exoenzymes is a fascinating evolutionary development in ants. We found that polyol production is a conserved trait among derived and less derived attinas. However, *Leucoagaricus gongylophorus*, the mutualistic fungus of the more derived leafcutter ants, expresses an incomplete set of enzymes necessary to reduce carbohydrates to polyols compared to *Leucocoprinus* sp., the mutualistic fungus of less derived ants. Although ants search for and consume these polyols with lower priority than glucose, polyols are important for developing and sporulating antagonistic fungi living in the fungus gardens. Therefore, despite the massive and widespread production by the fungus garden and the proposed benefits, polyols do not attract or preferentially feed the ants but stimulate invading fungi. Thus, the production of polyols was not a decisive factor in attracting ants to establish mutualism with fungi but rather in spreading fungi that can threaten ant colonies. Ants’ evolution mitigated this setback by increasing by-product diversity, minimizing polyol concentration, and switching to mutualistic fungi lacking the expression of critical enzymes for polyol biosynthesis.

**IMPORTANCE:**

Microbial communities are critical elements of biodiversity modulating life on Earth. Insects interact intensely with microorganisms to obtain nutrients. They also suffer from diseases caused by microbial infections. The present study is an example of how the different actors of a microbial community interact with each other and their insect hosts. We found that polyols produced by microbes that digest plant matter affect nutrition and facilitate infections in leafcutter ants. This knowledge is crucial for understanding ant-microbe interaction and controlling agricultural pest leafcutter ants.

## INTRODUCTION

Ants in the subtribe *Attina* live in North, Central, and mainly South America (1, 2). Attinas harvest various substrates to culture mutualistic fungi inside their nest. The fungus grows in the so-called fungus garden, digesting substrates to feed the ants (3, 4). This ancient mutualism thrived for 65 million years, leading to morphological and metabolic specialization and reciprocal benefits for both partners (5, 6).

The most derived attinas are the leafcutters in the genera *Atta*, *Acromyrmex*, *Amoimyrmex*, and *Pseudoatta* (6–9). *Atta sexdens* gardens digest plant polysaccharides into simple sugars that are crucial food sources for mutualists, ants (10), and fungi (4). Unexpectedly, *Atta bisphaerica* and its mutualistic fungus do not immediately consume most of the generated glucose and xylose, the more abundant monosaccharides produced in fungus gardens (11). Instead, the fungus garden microbes reduce these carbon sources to make large amounts of polyols, mannitol, and arabitol; the fungus garden also produces minor amounts of inositol and sorbitol; polyol production depends on microbial activity; and exudates found in the fungus garden are rich in polyols produced mainly by the mutualistic fungus, *Leucoagaricus gongylophorus*, and likely by other microbes (11).

The significance of polyol production is still enigmatic. Polyol accumulation could preserve the half-life of fungal depolymerase exoenzymes, responsible for digesting plant matter collected by ants and conserving surplus energy (11). Besides, polyols are reduced compounds rich in electrons, which could build an energetic stock for ants to consume in food scarcity situations like during winter.

Derived leafcutters cultivate the “domesticated” basidiomycete *Leucoagaricus gongylophorus,* which is distantly related to free-living fungi. In contrast, the less derived attinas cultivate fungi genetically similar to “wild-type” free-living species (5, 12, 13). Whether the strategy of accumulating mannitol and arabitol is exclusive to *Atta bisphaerica* gardens or found in other attinas is yet to be discovered. In addition, knowing whether mannitol and arabitol attract ants is essential for investigating the origins of ant and fungus mutualism. This putative attraction would have encouraged ant ancestors to collect and cultivate these fungi during the early stages of mutualism so that polyols could be an essential chemical signal of communication and recognition between mutualistic partners. Finally, the massive amount of polyols produced will likely affect other microorganisms in fungus gardens, such as antagonistic fungi (14–16).

In the present study, we tested four hypotheses: (i) polyols play a role in the communication between ants and mutualistic fungi; (ii) polyols are primary food sources for the ants; (iii) polyols production is a general feature in attine fungus gardens; (iv) polyols affect the microbial community living in fungus gardens. Our results contribute to decrypting polyol roles in ant-microbe interaction.

## MATERIALS AND METHODS

### Chemicals

Reagents used in the experiments were agar (Neogen Culture Media, Lansing, MI, USA), ammonium acetate (Sigma-Aldrich, A7262, USA), arabitol (Sigma-Aldrich, A3381, China), cellulose (Sigma-Aldrich, C6288, UK), glucose (Synth, 01G1008.01.AF, Brazil), inositol (Sigma-Aldrich, 17508, China), mannitol (Sigma-Aldrich, M4125, Brazil), sorbitol (Sigma-Aldrich, S1876, France), starch (Sigma-Aldrich, S 9765), xylitol (Sigma-Aldrich, X3375, USA), and yeast nitrogen base (Thermo Fisher Scientific, Q30009, USA).

### Fungus garden extracts

We collected portions of the fungus gardens of *Atta sexdens*, *Acromyrmex lundii,* and *Mycocepurus goeldii* and gently removed ants. Then, we added 5 mL of 0.1 M ammonium acetate to 1.0 g of each ant-free fungus garden sample and vortexed for 1 min at room temperature in a 15 mL sterilized Falcon tube. After centrifugation (8,000 rpm for 20 min), we filtered the supernatant (0.22 µm polyethersulfone, Kasvi membrane), lyophilized the filtrate, and stored it at −20°C. Typically, 1.0 g of fungus garden (wet weight) generated 0.26 g of lyophilized extract.

### Ant attractiveness test

**Fig 1:**
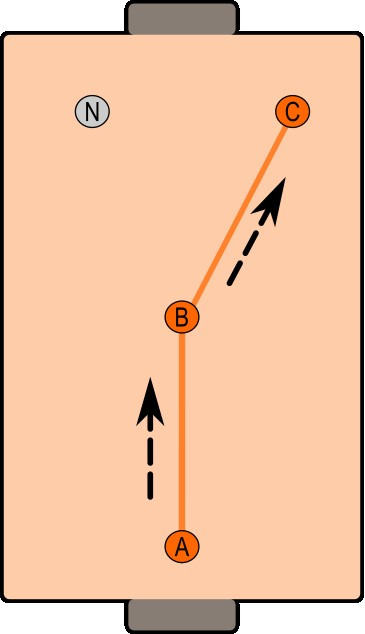
Schematic diagram of the trail choice test arena. Solid lines mean an existing track (200 µL compound solutions). Point A: location of added ants; point B: intersection; point N: no compound added; point C: addition of the test compound (50 µL of fungal garden extracts or 1 mol/L mannitol, arabitol, inositol, or sorbitol). The distance between the points was 15 cm.

We analyzed the attractiveness of fungus garden extracts and polyols on the leafcutters *Atta sexdens* and *Acromyrmex subterraneus,* as well as in the less derived attina *Mycocepurus goeldii*. We set up an experimental arena in a tray lined with filter paper (Fig. 1) (17, 18). Then, we removed the forager caste of ants from the colonies and placed them in a plastic pot for 10 min to reduce handling stress (19). Next, we gently placed each ant at the trail’s starting point (A) and observed it for 10 min. We used 15 ants per analyzed compound and 200 µL of 3.0 g/100 mL of *Acromyrmex subterraneus* fungus garden extract, 1.0 M polyol, or 1.0 M glucose, dissolved in ultrapure water to assemble the trails (solid lines connecting points A to B and B to C). Points A, B, and C received 50 µL extract or solutions each (Fig. 1). As the null treatment, we used ultrapure water.

### Carbon source consumption by ants

We evaluated the consumption of different food sources by *Atta sexdens*, filling a multiwell plate pit with 2 mL of 1.0 mol/L arabitol, inositol, mannitol, sorbitol, xylitol, or glucose diluted in ultrapure water. We placed food sources 10 cm from the colony entrance and independently tested 20 small young colonies, each with two replicates. We selected colonies collected in the field 30 to 60 days after the nuptial flight and brought and fed in the laboratory for 4 months. We interrupted the feeding 48 hours before the experiments to stimulate foraging. Each replicate simultaneously used all food sources and controls occupying different wells at randomly varying spatial positions in a multiwell plate, guaranteeing that ants experienced multi-choice situations. We used ultrapure and tap water as controls. After 24 hours, we collected and transferred the food remains with a pipette to a 0.1 mL graduated cylinder to calculate substrate consumption. No detectable evaporation occurred with polyol or glucose solutions in experimental controls without the ants, so the consumed substrate volume defined substrate preference. We detected 5% water evaporation and considered this value to calculate water consumption.

### Profiling of fungus garden metabolites

We resuspended 20 µg–111 µg lyophilized fungus garden extracts in 2.5 mL ultrapure water and injected 10 µL into a chromatograph to generate each extract’s metabolite fingerprints (11). The analyses used a high-performance liquid chromatography (HPLC) Shimadzu Prominence (Shimadzu, Kyoto, Japan) equipped with a refractive index detector (RID-10A, Shimadzu) and a Phenomenex Rezex RPM-Monosaccharide Pb^+2^ (8%) column (Phenomenex, Torrance, CA, USA). The analysis conditions were as follows: column temperature at 85°C, injection volume of 5 µL in an isocratic mobile phase (0.4 mL/min) of water. We prepared standard arabitol, inositol, mannitol, sorbitol, and xylitol, each at 0.50, 0.10, 0.15, 0.20, and 0.25 ng/mL to identify target polyols. We expressed concentration in milligrams of polyol per gram of fungus garden (wet weight) based on HPLC standard curves. We also performed a ^1^H-nuclear magnetic resonance (^1^H-NMR) analysis to confirm the presence of polyols. We performed NMR analyses using a Bruker (Billerica, MA, USA) DRX-400 instrument operating at 400 MHz and with samples dissolved in D_2_O. Because polyol produces a characteristic pattern of multiplicity and chemical shift in the NMR spectrum in a given solvent (20, 21), we used enlarged spectra to identify polyols.

### Microbial strains

The Molecular Evolution Laboratory provided the mutualistic fungus of leaf-cutting ants, *Leucoagaricus gongylophorus* strain B (22), mutualistic of *Atta sexdens*, and the mutualistic fungus of the less derived attina *Mycocepurus goeldii*, *Leucocoprinus* sp. We used these fungi to understand the effect of domestication on polyol production. The Laboratory of Fungal Ecology and Systematics at UNESP in Rio Claro provided the fungi living in the fungus garden, antagonists of the mutualistic fungus of ants: *Escovopsis* sp. (MOC201) (23), *Syncephalastrum* sp. (MOC87) (24), *Trichoderma* sp. (25); and the prevalent fungus living in the integument of leafcutters, *Cladosporium* sp. (MOC234) (26). We tested these fungi to understand the effect of polyol on the development of fungi that may harm the ants.

### Polyol pathways in mutualistic fungi

*L. gongylophorus* and *Leucocoprinus* sp. grew on yeast nitrogen base (Thermo Fisher Scientific Q30009) medium containing 1.0 g/100 mL starch as the sole carbon source, and Macrogen Inc. generated transcriptomic data using the HiSeq 2000 platform and 100 bp paired-end reads. FASTQC (http://www.bioinformatics.babraham.ac.uk/projects/fastqc/) determined quality and overrepresented sequences, Fast x_trimmer (http://hannonlab.cshl.edu/fastx_toolkit/index.html) cut the first 15 nucleotides of all reads, and SeqyClean (https://github.com/ibest/seqyclean) (27) trimmed low-quality raw reads (Phred Q score <30). Trinity (28) assembled high-quality sequences using digital normalization (29) to reduce sequence redundancy and improve transcript assembly, with 20× minimal coverage. BUSCO (30) evaluated the assembly completeness based on gene databases containing Fungi_odb10 universal single-copy orthologs (OrthoDB). CD-HIT-EST (31) minimized redundancy, applying a 90% similarity threshold. OmicsBox using BlastX (32) annotated genes with an E-value of 1e − 05 against the NCBI nonredundant database. We performed Gene Ontology functional classification into Biological Processes, Molecular Function, and Cell Component categories. InterProScan (33) found domain proteins, and OmicsBox attributed Enzyme Commission (EC) terms. We manually searched keywords to find enzymes involved with polyol production, examining gene lists for both fungal species based on literature searches for polyol metabolic pathways (Fig. 5). We also used known fungal sequences (Table 1) as queries in a BlastN using an e-value cutoff of 1e − 05 against the NCBI nonredundant database to identify enzyme transcripts. The identified enzyme transcripts (GenBank no. PV235367–PV235381, PV239755–PV239771 and PV395580–PV395584) of whole polyol biosynthetic pathways indicated the ability to produce polyols. Finally, we used OmicsBox to map transcripts of mutualistic fungi against genomic data (34) from *L. gongylophorus* cultured by *Atta colombica* and *Atta mexicana*.

**TABLE 1:** Mutualistic fungal genes coding for metabolic pathway enzymes converting cellulose and hemicellulose to polyols^*a*^

Enzyme						Transcriptome		Genome
Name	Short	EC no.	Kegg link	GenBank no.	GenBank link	*Leucocoprinus* sp.	*L. gongylophorus*	*L. gongylophorus*
Xylanase								
Endo-1,4-β-xylanase	Xylanase	EC:3.2.1.8	✓	XM_036677610	✓	Y	Y	N
Cellulases								
Endocellulases	Cellulase	EC:3.2.1.4	✓	NC_082405.1	✓	Y	Y	Y
β-Glucosidases		EC:3.2.1.21	✓	NW_023503004.1	✓	Y	Y	Y
Cellulose 1,4-beta-cellobiosidase		EC 3.2.1.91	✓	XM_037352492	✓	Y	Y	Y
Polyol biosynthesis pathways								
Aldose reductase	**ALR**	EC:1.1.1.21	✓	XM_046180420.1	✓	Y	N	Y
Xylitol dehydrogenase	**XDH**	EC 1.1.1.9	✓	XM_001386945	✓	Y	N	Y
Sorbitol dehydrogenase	SDH	EC 1.1.1.14	✓	XM_014328970	✓	Y	Y	Y
Mannitol dehydrogenase	MtDH	EC 1.1.1.138	✓	XM_046180790	✓	Y	Y	Y
Mannitol 1-phosphatase	M1Pase	EC:3.1.3.22	✓	NZ_CP013018.1	✓	N	N	N
Mannitol-1-phosphate 5-dehydrogenase	M1PDH	EC:1.1.1.17	✓	XM_007370098	✓	Y	Y	Y
Glucose-6-phosphate isomerase	PGI	EC:5.3.1.9	✓	XM_069349660	✓	Y	Y	Y
Hexokinase	Hk	EC 2.7.1.1	✓	XM_001878852.1	✓	Y	Y	Y
Inositol monophosphatase	IMPase	EC:3.1.3.25	✓	XM_067676709.1	✓	Y	Y	Y
Inositol-3-phosphate synthase	MIPs	EC:5.5.1.4	✓	XM_062770731	✓	Y	Y	Y
Non-oxidative polyol production pathway								
Transketolase	TKL	EC:2.2.1.1	✓	XM_062770731	✓	Y	Y	N
Ribulose-phosphate 3-epimerase	RPE	EC:5.1.3.1	✓	XM_069352554	✓	N	Y	Y
Oxidative polyol production pathway								
UDP-glucose 6-dehydrogenase	UDGH	EC:1.1.1.22	✓	XM_060268098	✓	Y	Y	Y
6-Phosphogluconolactonase	6 PGL	EC:3.1.1.31	✓	XM_001873856	✓	Y	Y	Y
Phosphogluconate dehydrogenase	6PDGH	EC:1.1.1.44	✓	XM_031171234	✓	Y	Y	Y
Ribulokinase	RK	EC:2.7.1.16	✓	XM_033591120	✓	N	N	N
Xylulokinase	XK	EC:2.7.1.17	✓	XM_016418222	✓	Y	Y	Y
Arabitol −4 dehydrogenase	**Ar4DH**	EC:1.1.1.11		–	✓	Y	N	Y
Arabitol −2 dehydrogenase	**Ar2DH**	EC:1.1.1.250	✓	XM_064908036	✓	Y	N	Y

^*a*^ –, not found. ✓, Kegg or GenBank link. Bold, missing in *Lecoagaricus gongylophorus*, but found in *Leucocoprinus* sp. Y, gene or gene expression was detected; N, no gene or gene expression was detected.

### Fungal growth and sporulation tests

We cultured each fungus in Petri dishes containing solid culture media (1.5% agar), 0.5 mol/L of polyol (mannitol, inositol, or sorbitol), 171.15 g/L cellulose, or 0.65 g/L of *Acromyrmex subterraneus* fungus garden extract. We used water agar (1.5% agar) as negative fungal growth control, representing the absence of a carbon source, and potato dextrose agar (PDA) as a positive growth control of culture viability. We inoculated 3 µL of a 5 × 10^4^/mL spore suspension on the culture media in the Petri dish center. After incubation at 25°C for 12 days, we photographed the plates to measure the fungal colony diameter, which varied from 0 up to 8 cm, corresponding to the total diameter of a Petri dish. The polyols tested were mannitol, sorbitol, and inositol because we found their biosynthetic pathways in mutualistic fungi. Additionally, we used extracts from the fungal garden of *A. subterraneus*, cellulose, and glucose because these are common nutrients available in the fungus garden to ensure the practical relevance of our research. We tested each substrate in triplicate.

### Statistical analyses

We performed all the analyses on R software version 4.3.0 (35). To test fungal growth, we used the analysis of variance test. The halo size of fungal growth (ranging from 0 to 8 cm) served as the response variable, while the carbon source was the explanatory variable. For pairwise comparisons, we used the Tukey test. To study substrate consumption by the ants, we applied a generalized linear model using the family quasipoisson because our data showed overdispersion (36). We applied a model that considered consumption as the response variable, with carbon source and concentration as the explanatory variables. Additionally, we estimated marginal means (EMMs) tests using the emmeans package (version 1.8.6) (37). For pairwise comparisons, we employed the robust “fdr” correction.

## RESULTS

### Attractivity and consumption of polyols by ants

**Fig 2:**
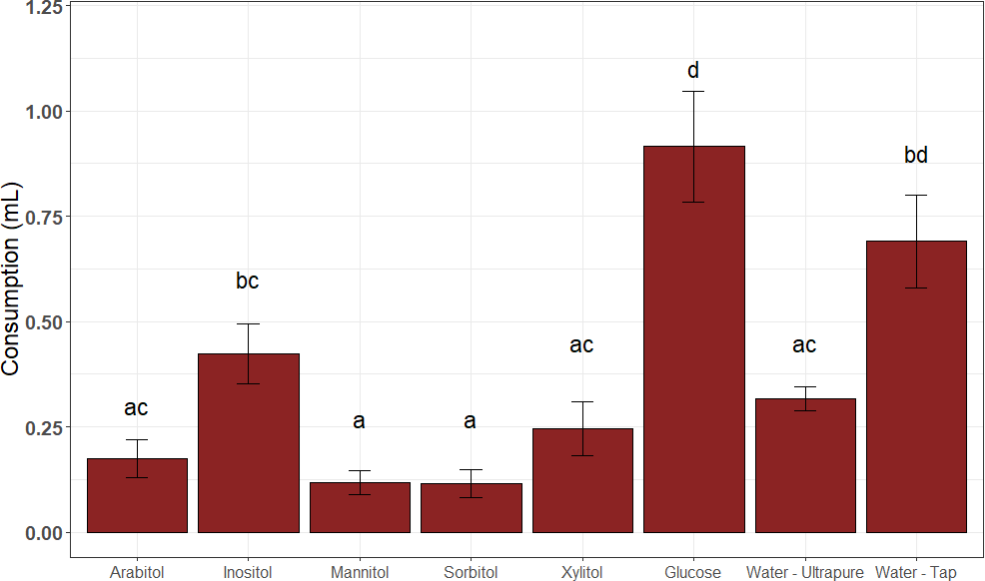
Ant consumption of substrates. Bars indicate standard error. According to the emmeans test, bars with distinct letters differ significantly (*P* < 0.01).

Polyols or fungus garden extracts did not attract the ants, which did not follow polyol trails. The ants in the test arena ignored the trails and walked randomly or groomed themselves. On the other hand, consumption analysis indicated a preference for glucose (*P* < 0.01) over inositol, xylitol, arabitol, mannitol, and sorbitol (Fig. 2) and a preference for inositol over mannitol and sorbitol. Thus, the ants did not preferentially consume mannitol and arabitol, the highest-produced polyols in the fungus garden preparations (11). During the experimental time, tap water was more consumed than ultrapure water.

### Metabolite profiling

**Fig 3:**
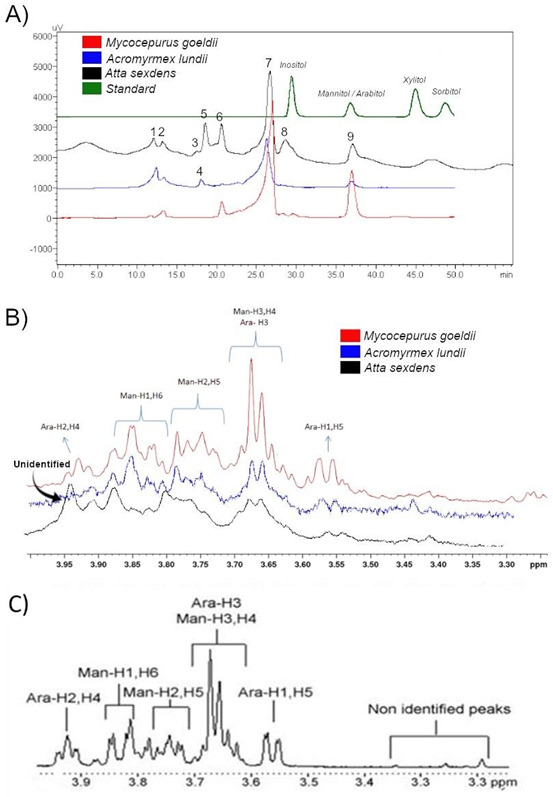
Metabolite fingerprints. (**A**) Chromatograms of fungus garden extracts from three colonies of different ant species. *Mycocepurus goeldii*, which cultivates *Leucocoprinus* sp., *Atta sexdens,* and *Acromyrmex lundii,* which cultivate *Leucoagaricus gongylophorus*. Chromatograms presented baselines at 0 mV and were artificially dislocated in the *y*-axes for better visualization. Standard polyols were at 0.25 ng/mL each. The retention times of mannitol and arabitol (peak 9) was 37.030 min and identified metabolites compatible with mannitol and arabitol in the fungus gardens, in higher concentration in *M. goeldii* than in *Atta sexdens* or *Acromyrmex lundii* fungus gardens. (**B**) ^1^H NMR enlarged spectra obtained from the fungus garden extract of *Mycocepurus goeldii*, *Acromyrmex lundii,* or *Atta sexdens* in D_2_O. Note the pattern of multiplicity and chemical shift characterizing arabitol and mannitol. The Ara-H2,H4 signature only in *M. goeldii* fungus garden extract, indicating that arabitol was detected in *M. goeldii* but not in *Acromyrmex lundii* or *Atta sexdens* fungus garden; Man-H1,H6 and Man-H2,H5 were in both *M. goeldii* and *Acromyrmex lundii* and residual in *Atta sexdens*; Man-H3,H4/Ara-H3 and Ara-H1,H5 were in *M. goeldii* and residual in *Acromyrmex lundii* and *Atta sexdens*, indicating mannitol is at higher concentration in *M. goeldii* than in *Acromyrmex lundii* or *Atta sexdens* fungus gardens. These results indicate both mannitol and arabitol in the fungus garden extract of *Mycocepurus goeldii* and only mannitol in the fungus garden extracts of *Acromyrmex lundii* or *Atta sexdens*. (**C**) ^1^H NMR enlarged spectra, showing the characteristic signatures of mannitol and arabitol dissolved in D_2_O (20).

We characterized distinct chromatographic fingerprints for each fungus garden (Fig. 3A). The more derived *A. sexdens* fungus garden showed eight peaks (named 1, 2, 3, 5, 6, 7, 8, and 9); the *Acromyrmex lundii* fungus garden generated five peaks (1, 2, 4, 7, 9); and the less derived *M. goeldii* fungus garden presented four peaks (2, 6, 7, and 9). These results indicate that the diversity of metabolites in fungus gardens increased with the evolution of ants.

Peaks 1, 2, 7, and 9 were present in the fungus gardens of the three ant species; peaks 3, 5, and 8 were exclusive to *A. sexdens*; and peak 4 was exclusive to *A. lundii*. Exclusive peaks indicate metabolic specializations characteristic of leafcutter ants, while the absence of these exclusive peaks indicates the ancestral state retained in the less derived ant *M. goeldii*.

Peak 9 was the only one showing retention time compatible with standard polyols, co-eluting with mannitol and arabitol standards. NMR spectra (Fig. 3B) presented the typical carbohydrate signals (20, 21) at 3.5 ppm–4.0 ppm, which comprehend the polyol region and identified both mannitol and arabitol in the fungus garden extract of *Mycocepurus goeldii* and only mannitol in the fungus garden extracts of *Acromyrmex lundii* or *Atta sexdens* (Fig. 3B). Therefore, concentration in the neoattine fungus garden could be calculated as 0.276 for *Acromyrmex lundii* and 0.250 mg/g (wet weight of fungus garden) for *Atta sexdens*, assuming only mannitol is present in neoattines peak 9. NMR mannitol signal (and, therefore, concentration) was also lower in *Atta sexdens* than in *Acromyrmex lundii* garden and even lower when compared with the garden of *Mycocepurus goeldii*. Thus, more derived ants show a decrease in the mannitol concentration of fungus gardens compared to less derived ants. These results indicate that ant evolution decreased fungus garden polyol production.

### Polyol pathway identification

**Fig 4:**
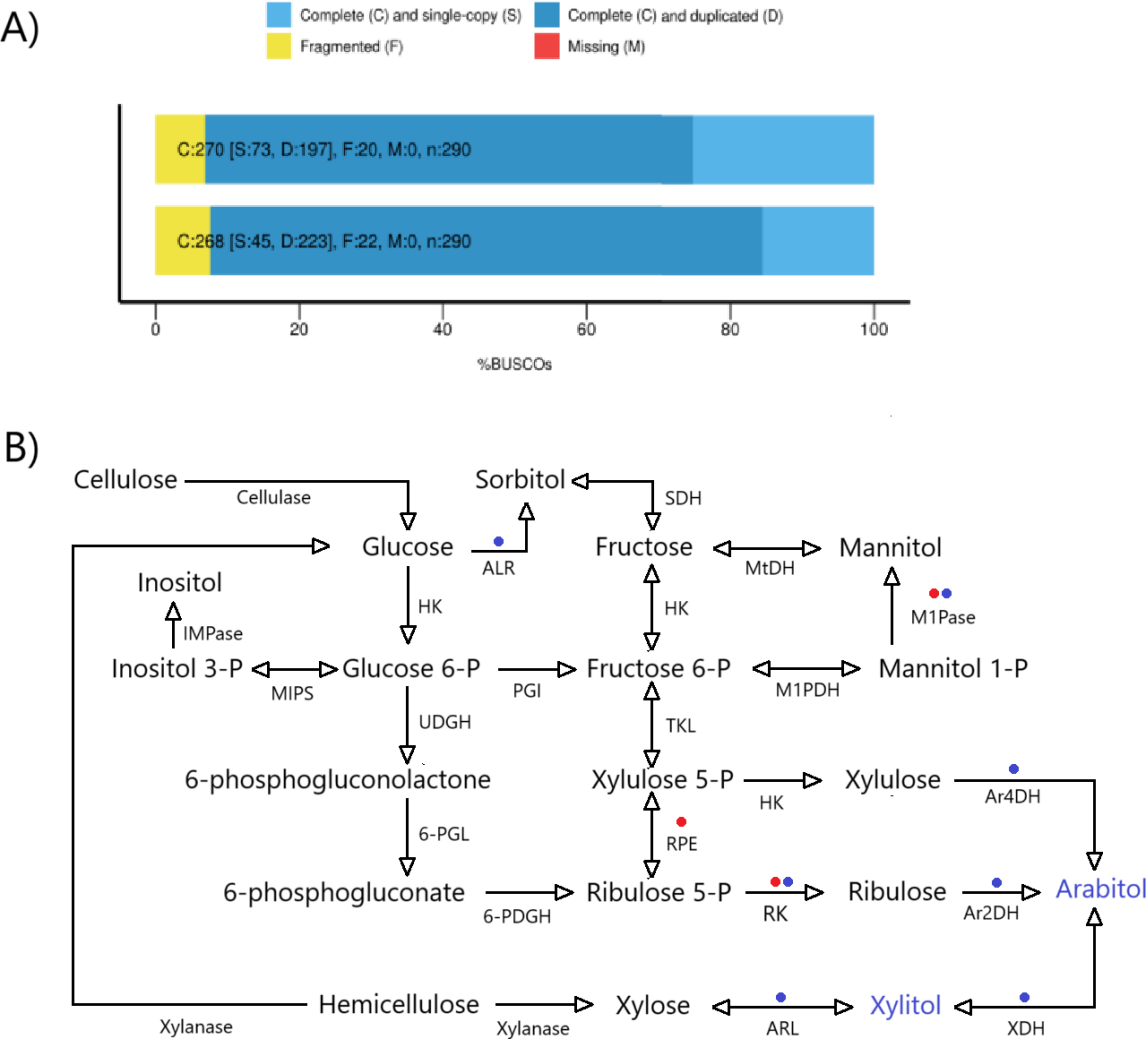
BUSCO assessment results for *Paleoattina* (*Leucocoprinus* sp.) or *Neoattina* (*Leucoagaricus gongylophorus*) mutualistic fungi (**A**). Polyol metabolic pathways in *Paleoattina* or *Neoattina* mutualistic fungi based on transcriptomic data (**B**). We assembled fungal polyol pathways (39–45) and looked for each enzyme transcript in mutualistic fungi. The circle indicates a lack of transcript in the *Paleoattina* fungus mutualist *Leucocoprinus* sp. (red) or *Neoattina* mutualist *Leucoagaricus gongylophorus* (blue). Xylanase (endo-1,4-β-xylanase [EC:3.2.1.8]); xylitol dehydrogenase, XDH (EC:1.1.1.9); cellulase (endocellulases, [EC:3.2.1.4]; β-glucosidases [EC:3.2.1.21]; cellulose 1,4-beta-cellobiosidase [EC:3.2.1.91]); aldose reductase, ALR (EC:1.1.1.21); sorbitol dehydrogenase, SDH (EC:1.1.1.14); mannitol dehydrogenase, MtDH (EC 1.1.1.138); mannitol 1-phosphatase, M1Pase (EC:3.1.3.22); mannitol 1-phosphate 5-dehydrogenase, M1PDH (EC:1.1.1.17); glucose 6-phosphate isomerase, PGI (EC:5.3.1.9); hexokinase, HK (EC:2.7.1.1); inositol monophosphatase, IMPase (EC:3.1.3.25); inositol 3-phosphate synthase, MIPs (EC:5.5.1.4); non-oxidative pentose phosphate pathway (PPP) (transketolase, TKL [EC:2.2.1.1]; ribulose-phosphate 3-epimerase, RPE [EC:5.1.3.1]); oxidative PPP (UDP-glucose 6-dehydrogenase, UDGH [EC:1.1.1.22]; 6-phosphogluconolactonase, 6PGL [EC:3.1.1.31]; phosphogluconate dehydrogenase [NADP^+^-dependent, decarboxylating], 6PDGH [EC:1.1.1.44]); ribulokinase, RK (EC:2.7.1.16); ribulose phosphate 3-epimerase, RPE (EC:5.1.3.1); xylulokinase, XK (EC:2.7.1.17); arabitol-4 dehydrogenase, Ar4DH (EC:1.1.1.11); arabitol-2 dehydrogenase, Ar2DH (EC:1.1.1.250). GenBank accession numbers for transcripts are PV235367–V235381, PV239755–PV239771, and PV395580–PV395584. Note that *Leucoagaricus gongylophorus*, the fungus mutualistic with more derived leafcutters, did not express ALR, XDH, Ar2DH, and Ar4DH, necessary to produce xylitol and arabitol (highlighted in blue). However, *Leucocoprinus* sp., the fungus symbiotic with the Paleoattina *Mycocepurus goeldii*, expressed these four genes.

We got a high-quality gene assembly for either *Leucocoprinus* sp. or *Leucoagaricus gongylophorus*, as most gene sequences corresponded to complete fungal sequences (Fig. 4A). Transcriptomic data recovered over 150 million filtered reads (Table 2), which is needed to detect expressed genes (38). We reconstructed polyol biosynthetic pathways based on the literature on fungal metabolism and found transcripts for xylitol, arabitol, sorbitol, mannitol, and inositol biosynthesis in *Leucocoprinus* sp. cultured on starch. However, in *Leucoagaricus gongylophorus* cultured on starch, we only found transcripts for sorbitol, mannitol, and inositol biosynthesis (Fig. 4B). Therefore, *L. gongylophorus* cultures did not express many of the enzymes for polyol biosynthesis found in *Leucocoprinus* sp. cultures, including ALR, XDH, AR2DH, and AR4DH, which are necessary for xylitol and arabitol biosynthesis (Fig. 4A). Finally, in the genome (34) of *L. gongylophorus,* mutualistic with *Atta colombica* or *Atta mexicana*, we found ALR, XDH, AR2DH, and AR4DH genes (Table 1).

**TABLE 2:** Transcriptome data obtained from laboratory cultures of ant mutualistic fungi

Parameter	Fungal symbiont isolate	
	*Leucoagaricus gongylophorus*	*Leucocoprinus* sp.
Base pairs	16,661,050,092	15,682,369,384
Filtered reads	164,960,892	155,270,984
Q30 (%)	89.28	88.35
Contigs	56,725	90,451
Average contig size	2,062.89	1,653.88
GC content (%)	46.87	48.95
Contigs over 300 bp	53,718	82,940

### Fungal growth and sporulation

**Fig 5:**
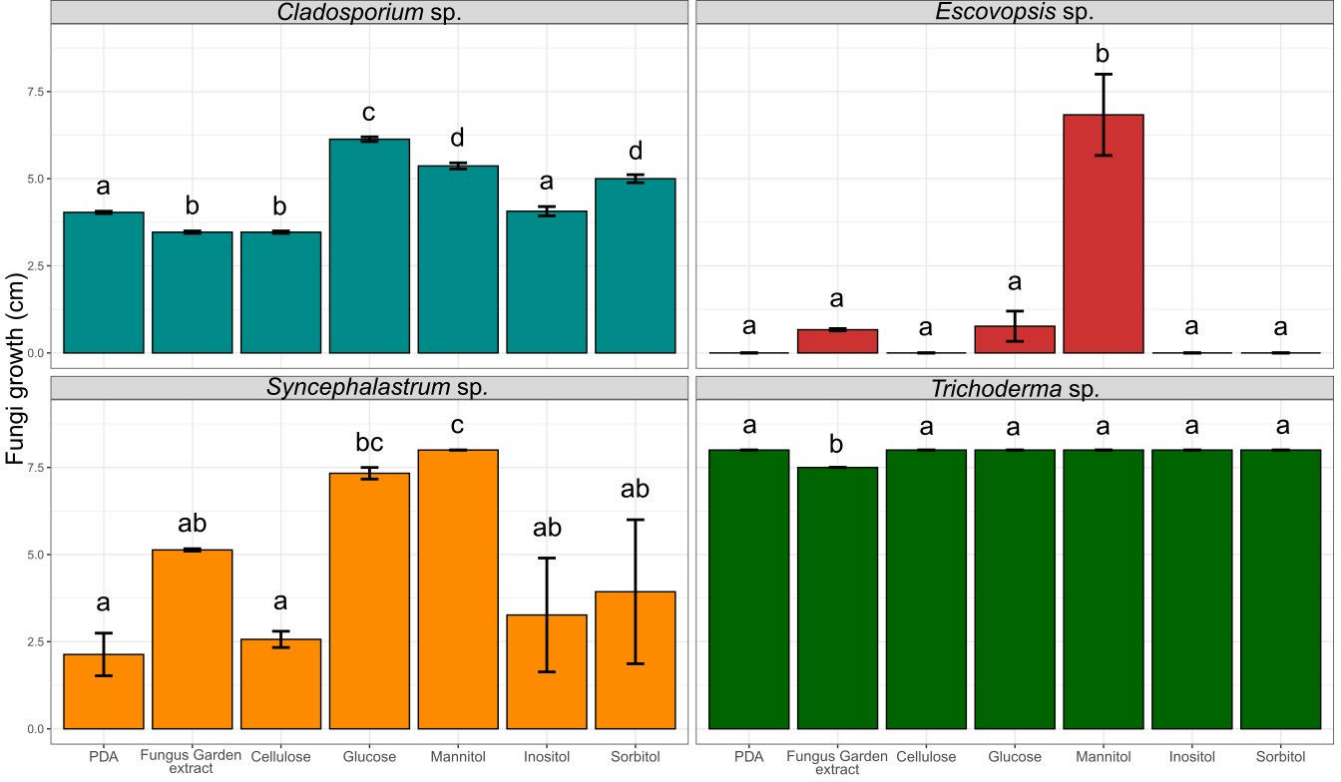
Growth of antagonistic fungi in different carbon sources. Bars with distinct letters are significantly different (*P* < 0.01), according to Tukey’s test.

Unlike ants, which prefer glucose over polyols, antagonistic fungi prefer mannitol. It was within the best carbon sources supporting the growth of all tested fungi and the only one supporting significant *Escovopsis* sp. growth (Fig. 5). Differently from *Escovopsis*, which specializes in assimilating mannitol, *Cladosporium* sp., *Syncephalastrum* sp., and *Trichoderma* sp. are more flexible generalists, assimilating carbons from polyols, glucose, cellulose, and fungus garden extract (Fig. 5).

Regardless of the carbon source, *Trichoderma* sp. never sporulated, as *Syncephalastrum* sp. always sporulated (Table 3). On the other hand, some fungi may sporulate depending on the carbon source: *Cladosporium* sp. on inositol and *Escovopsis* sp. on mannitol (Table 3). Therefore, polyols are important for sporulation in *Cladosporium* sp. and *Escovopsis* sp.

**TABLE 3:** Fungal sporulation on different carbon sources^*a*^

Substrate	Fungus			
	*Syncephalastrum* sp.	*Escovopsis* sp.	*Trichoderma* sp.	*Cladosporium* sp.
Fungus garden extract	++	−	−	++
Cellulose	++	−	−	−
Glucose	+	−	−	−
Mannitol	+	+	−	−
Inositol	+	−	−	+
Sorbitol	+	−	−	−
PDA	+	−	−	−

^*a*^ Sporulation typically occurs in clusters of aerial hyphae distributed throughout the Petri dish. We classified it as (+). When sporulation intensifies, we also noted spores in the hyphae that grow on the surface of the culture medium. So, when aerial and surficial hyphae sporulated, we classified it as (++). (−) indicates no sporulation. The results were consistently obtained in every triplicate.

## DISCUSSION

Polyol is a known fungal by-product with many implications for fungal life (46). It is detected in the environment as a marker of fungal activity (47). So, Somera et al.’s (11) first report of polyol in the fungus garden was not unexpected; still, the effects of massive polyol production by the leafcutter fungus garden (11) still need to be studied.

Given the putative biological importance of these fungal by-products, we expected the ants’ behavior to respond to polyols. Thus, one hypothesis tested in the present investigation was whether polyols play a role in the communication between ants and mutualistic fungi. So, we tested whether polyols attract ants, similarly to pheromones, which trigger ants to follow trails left by their colony mates (17). Since ants can discriminate and follow trail pheromones (48, 49), polyol production could have played a part in ants’ initial recognition and further communication with putative fungal partners. However, this was not the case since artificial trails built with polyols or fungus garden extract did not attract the ants. Thus, unlike pheromones, polyols do not trigger ant following behavior. Therefore, polyols’ function relies exclusively on ant nutrition, with no role in mutualist communication.

Indeed, our experiments demonstrate that ants actively fed on polyols (Fig. 2 and 3), which agrees with the findings that leafcutters consumed polyols produced inside the colony (11). Still, glucose was the highest-preferred food source, reinforcing the proposal (10) that glucose produced in the fungus garden is the most crucial carbon source for feeding the ants. Therefore, polyols are secondary food sources and may alternatively feed ants in nutrient scarcity situations, for instance, during winter.

We found that mannitol production is a conserved and likely general feature in attine fungus gardens and that mannitol production was higher in less derived attines (Fig. 3B). However, arabitol production was present in less derived but not in more derived attine fungus gardens. Thus, ant evolution minimized mannitol and arabitol production in the fungus garden.

In addition, each garden had a specific HPLC fingerprint for catabolite production (Fig. 3A), revealing specializations in metabolite production. Increasing fingerprint complexity shows that ant evolution resulted in a diversification of fungus garden by-products.

In agreement with this diversification, we revealed anabolic pathways for polyols in mutualistic fungi associated with ants (Fig. 4B). We successfully identified transcripts related to enzymes involved in the anabolic routes of xylitol, sorbitol, mannitol, arabitol, and inositol in cultures of mutualistic fungi isolated from the *Paleoattina* fungus garden, so we pictured complete pathways leading plant cellulose and hemicellulose carbons to polyols in this fungus. (Fig. 4B). However, cultures of the fungus mutualistic with *Neoattina* do not express crucial enzymes for xylitol and arabitol biosynthesis. The lack of expression may reflect transcriptional suppression once the corresponding genes are present in the *L. gongylophorus* genome (34) (Table 1). Suppression may also occur in the ant nest once arabitol was not detected in the fungus garden (Fig. 3).

Somera et al. (11) and our current investigation (Fig. 3) detected mannitol production in the fungus garden of *Neoattina* culturing *Leucoagaricus gongylophorus*. We found transcripts (Fig. 4) coding for essential enzymes for mannitol biosynthesis in laboratory cultures of the isolated *L. gongylophorus*. Therefore, we can confirm that isolated *L. gongylophorus* can produce mannitol. However, although Somera et al. (11) detected arabitol production in the leafcutter *Atta bisphaerica* fungus garden, our ^1^H-NMR spectra indicate that the fungus garden of the leafcutter species *Atta sexdens* and *Acromyrmex lundii* do not produce arabitol. This finding and differences in HPLC fingerprints of leafcutters (Fig. 3A) indicate that the fungus garden’s production of arabitol and other metabolites may vary among different leafcutter species.

Since *L. gongylophorus* isolates from different leafcutters are nearly genetically identical over a wide geographic range (22), it seems unlikely that arabitol production varies drastically among isolates. However, another microbe symbiont may produce arabitol in the fungus garden (50) once various microbes synthesize compounds within the ant colony (51).

We also tested whether the massive amounts of polyols affect the microbial community living in fungus gardens. We found that mannitol is the most important carbon source supporting the development of antagonistic fungi and that polyols are essential for *Cladosporium* sp. and *Escovopsi*s sp. to sporulate. Mannitol is important for *Escovopsis* to sporulate, similar to *Stagonospora nodorum,* which causes the “Glume Blotch” disease in wheat crops (39). *Stagonospora nodorum* produces mannitol from plant glucose or fructose (52). Conversely, antagonistic fungi do not need to synthesize mannitol; they can obtain mannitol directly from the fungus garden, which makes sporulation less costly than in *S. nodorum*. Thus, *Escovopsis* sp. likely tracks mannitol produced in the fungus garden to thrive in the attina nests. Likewise, mannitol favors *Cladosporium* sp. proliferation. Therefore, polyol production by the fungus garden exposes the ants to unwanted fungi. Considering that mannitol protects parasitic fungi against the chemical defenses of parasitized plants (53), it is conceivable that attina fungal parasites benefit from similar protection. Finally, once antagonists assimilate polyols, they compete with ants and the mutualistic fungus for carbon sources. These results indicate that fungal garden antagonists benefit from polyol production by the fungus garden. Therefore, adaptation to a polyol-rich environment may have improved the antagonist’s fitness to attina fungus garden.

Our data show that polyols and glucose represent distinct nutritional niches. Ants make their decisions based on the availability of glucose, which is more nutritious for them. On the other hand, polyols are more critical for fungi, notably mannitol. Mannitol rivals glucose as a carbon source for growth, is important for sporulation, and improves the development and spreading of antagonistic fungi. As the mutualist fungus uses glucose to produce mannitol, it drains vital nutritional resources from the ants and facilitates infection.

Mannitol accumulation is an underlying threat to mutualism, given that the production and use of polyols are a general fungal trait. Leafcutter ants seem to have minimized this threat by selecting fungus gardens that diversified metabolite production, which diverted carbons that could produce mannitol, mitigating infection by antagonistic fungi. Thus, as mutualistic fungus domestication occurred, ants reduced infection risks by draining carbons from mannitol biosynthesis. Finally, cultures of the mutualistic fungus of *Paleoattina*, but not *Neoattina*, expressed enzymes for mannitol biosynthesis. It means that the evolution of attinas selected a fungus with reduced mannitol production, fighting antagonistic fungi stimulation.

The leafcutter mutualistic fungus, *Leucoagaricus gongylophorus*, presents low genetic diversity (22), seems younger than leafcutters (54), and is associated not only with the more derived *Atta* and *Acromyrmex* leafcutters but also with the distantly related less derived attina, *Apterostigma megacephala* (55, 56). The rapid spreading of *Leucoagaricus gongylophorus* within attinas indicates high ecological success and specialization. Still, it makes ants more susceptible to mycophagous infections since they have come to depend on a single fungal species. Therefore, reducing infection risk by lowering polyol production may have contributed to *L. gongylophorus* ecological success.

## Data Availability

Transcriptome data are available in the National Center for Biotechnology Information (NCBI) database: BioProject (PRJNA1215297), BioSample (SAMN46401056–SAMN46401057), and GenBank (PV235367–PV235381, PV239755–PV239771, and PV395580–PV395584).
